# Unexplained haemorrhagic fever in Rural Ethiopia

**DOI:** 10.11604/pamj.supp.2017.27.1.12567

**Published:** 2017-05-28

**Authors:** Zegeye Hailemariam, Doreen Tuhebwe, Meeyoung Mattie Park, Casey Daniel Hall

**Affiliations:** 1 Ethiopia Field Epidemiology Training Program, Addis Ababa, Ethiopia; 2 Uganda Field Epidemiology Training Program, Kampala, Uganda; 3 Rollins School of Public Health, Emory University, Atlanta, USA

**Keywords:** Public health, epidemiology, Ethiopia, haemorrhagic fever, outbreak investigation

## Abstract

This case study was written based on events of an outbreak investigation of an unfamiliar disease in Ethiopia during October–December 2012. Ethiopia did not have reports of similar cases in the 50 years prior to this outbreak. In this case study, we recapitulate and analyse this outbreak investigation based on data gathered from the community, health facility, and laboratory systems. It can be used to teach: 1) the outbreak investigation process; 2) selection of appropriate epidemiological design for the investigation process, 3) basic statistical analysis of surveillance data, and 4) principals of disease control. The target audiences for this case study are officials working in public health and public health trainees. It will take at most 3.5 hours to complete this case study. At the end of the case study, participants should be able to apply the principals of outbreak investigation and use surveillance data to respond to an outbreak in their country-specific context.

## How to use this case study

General instructions: to conduct this case study in the classroom, the authors propose that the participant’s guide be distributed one part at a time. Background material, including the appendices, should be distributed before the case study for review by the participants. Participants should take turns reading the narrative aloud, paragraph by paragraph. Reading all paragraphs aloud and in turns has two advantages: first, everyone is given an opportunity to participate and get beyond the inhibition of having her/his voice heard in a large room; second, the whole class is given time to understand the issue and think about the answers. The participants reading the question may try to answer it if s/he can; otherwise, it can be discussed as a group or completed as an exercise as the instructor’s notes dictate. Instructor’s notes are included in the instructor’s guide for each question. Complete all reading and questions before the next part is distributed. Then the next participant continues and so on until the case study is over. Once the conclusion is read, re-visit the learning objectives – this reinforces the learning and provides an opportunity to clarify any remaining issues.

**Audience:** officials working at the National Surveillance Department, Ministry of Health, Regional Surveillance Office, District Surveillance Office, District Head of Heath Department, and FETP/ Public Health trainees.

**Prerequisites:** before using this case study, participants should have received lectures in outbreak investigation, application of epidemiological study designs, and Integrated Disease Surveillance & Response guidelines and have experience or be currently working in a health-related field, or contributing to the government health surveillance functions.

**Materials needed:** white board or flip chart and marker

**Level of training and associated public health activity:** intermediate – outbreak investigation

**Time required:** approximately 3.5 hours

**Language:** English

## Case study material

- Download the case study student guide (PDF - 2.33 MB)
- Request the case study facilitator guide

## References

[1] WHO, CDC Technical Guidelines for Integrated Disease Surveillance and Response in the African Region.

[2] Ethiopian Health and Nutrition Research Institute Public Health Emergency Management Guideline.

[3] Gordis L (2013). Epidemiology.

[4] De Luca D’Alessandro E, Giraldi G (2011). A world wide public health problem: the principal re-emerging infectious diseases. Clin Ter.

[5] Onyango CO, Grobbelaar AA, Gibson GVF, Sang RC, Sow A, Swaneopel R (2004). Yellow fever outbreak, Southern Sudan, 2003. Emerg Infect Dis.

[6] WHO (2011). Revised recommendations for yellow fever vaccination for international travellers, 2011. Wkly Epidemiol Rec.

[7] Park MM, Hall CD, Frimpong JA, Dacuan L (2016). Case Study Development Course - Types of Experimental and Observational Epidemiologic Studies.

[8] WHO

[9] Kebede S, Duales S, Yokouide A, Alemu W (2010). Trends of major disease outbreaks in the African region, 2003-2007. East Afr J Public Health.

[10] Wiysonge CS, Nomo E, Mawo J, Ofal J, Mimbouga J, Ticha J (2008). Yellow fever control in Cameroon: where are we now and where are we going?. BMC Med.

[11] Aseffa A (1993). Viral diseases in Ethiopia: a review. East Afr Med J.

[12] Ethiopia Ministry of Health (2013). Recurrence of Yellow Fever-South Omo Zone, Southern Nationalities and Peoples. Ethiopia.

[13] Whitehead SS, Blaney JE, Durbin AP, Murphy BR (2007). Prospects for a dengue virus vaccine. Nat Rev Microbiol.

